# From barriers to participation: co-creating an effective reminder letter for breast cancer screening among underserved women in Flanders

**DOI:** 10.1186/s13690-025-01591-7

**Published:** 2025-05-14

**Authors:** Allegra Ferrari, Liesbet Van Bos, Sarah Talboom, Wessel van de Veerdonk, Wendy D’haenens, Marina Pak, Marlies Descan, Stephanie Parmentier, Louise Van Collie, Pascale Sibiet, Mathieu Goossens, Guido Van Hal

**Affiliations:** 1 Social Epidemiology and Health Policy (SEHPO), University of Antwerp, Antwerp, Belgium; 2 Department of Health Sciences, University of Genoa, Genoa, Italy; 3 Centre of Expertise - Care and Well-Being, Thomas More University of Applied Sciences, Mechelen, Belgium; 4 Centre of Expertise - Sustainable Business and Digital Innovation, Thomas More University of Applied Sciences, Mechelen, Belgium; 5 Federatie Van Mondiale & Democratische Organisaties (FMDO), Ostend, Belgium; 6 ENTER Project Working Group, Bruges, Belgium; 7 Lokaal Gezondheidsoverleg (Logo) Logo Brugge-Oostende Vzw, Bruges, Belgium; 8 Stad Oostende, Ostend, Belgium; 9 Center for Cancer Detection (Cvko), Bruges, Belgium

## Abstract

**Background:**

With over 2 million cases diagnosed annually, breast cancer is a leading cause of cancer-related disability and mortality worldwide. Despite global efforts to implement screening programs, uptake rates vary widely across settings due to socioeconomic factors and accessibility challenges. In 2022 in Flanders (Belgium), breast cancer screening participation in municipalities with an income below the poverty line was 15% lower than average.

**Methods:**

To tackle the limited participation of underserved women in the breast cancer screening program in Flanders, a culturally sensitive approach was used to investigate factors influencing screening participation and to realize a tailored reminder letter to be tested in a later phase. Working closely with community organizations, 33 women aged 50–69 (29 of whom were non-native Dutch speakers) with low-socioeconomic status were identified to participate in the study. Through an iterative process comprising 3 focus group discussions, 3 Delphi-consultations with sector experts, 1 co-creation session and a final member check, critical insights were gathered.

**Results:**

Key barriers included low health literacy and limited understanding of preventive care concepts. Once participants were effectively informed about the breast cancer screening program, they displayed increased help‐seeking behaviors in relation to health, underscoring the importance of clear communication in fostering willingness to consider screening. An evaluation of the standard invitation letter employed in the program revealed several challenges related to readability and comprehension. These included the excessive text length, the use of complex vocabulary and grammar beyond an A2 level, slogans unrelated to the mammography appointment (e.g., ‘We do it and what do you do?’), and the use of generic visuals. At the same time, simplifying the vocabulary to A1-A2 levels, employing straightforward sentence structures, and incorporating relevant visuals enhanced understandability and fostered interest in breast cancer prevention. Utilizing a color palette associated with breast cancer and featuring logos of local authorities instilled a sense of credibility and trustworthiness. Based on this feedback, a revised reminder letter was developed. The final communication was concise and included essential details such as time and place for screening and a QR code providing translation into 12 languages.

**Conclusions:**

Simplifying vocabulary, grouping related information, and providing direct links and language options improved the clarity and accessibility of the reminder letter, thereby fostering help‐seeking behaviors related to breast cancer screening.

**Supplementary Information:**

The online version contains supplementary material available at 10.1186/s13690-025-01591-7.

**Table:** 

Text box 1. Contributions to the literature
• This study provides valuable insights into the barriers to breast cancer screening participation among underserved women in Flanders (Belgium), a topic with limited existing research in this local context.
• Demonstrates the importance of culturally tailored communication strategies to enhance participation in public health programs.
• Provides a replicable model for redesigning communication materials, emphasizing simplicity, clarity, and trust-building visuals.
• Highlights the value of iterative co-creation with underserved communities to develop interventions that are both practical and culturally sensitive.
• Offers evidence on the usefulness of a tailored reminder letter in increasing help-seeking behaviors related to breast cancer screening, with potential policy implications for implementation.

## Introduction

### Background

Breast cancer (BC) ranks as the second most common tumor overall and the most common among women, with over two million cases diagnosed globally each year [1]. In 2019 it was the foremost cause of cancer-related disability-adjusted life years (DALYs) (20.3 million) and mortality (689,000) among women worldwide [2].

In 2022, Belgium exhibited one of the highest age-standardized incidence rates of breast cancer globally, at 104.4 per 100,000, which resulted in 2,324 related deaths [1]. Notably, the incidence rate in Flanders is even higher at 105.3 per 100,000, making it the region with the second highest age-standardized incidence rate worldwide after France [3].

Screen-detected breast cancers identified through screening mammography are generally at earlier stages, better differentiated, less likely to have spread, and have lower proliferation rates, leading to more favorable prognoses, compared to those found by other means [4, 5]. A recent study has found that the breast cancer screening program (BCSP) in Flanders can reduce BC-specific mortality by 51% [6].

Suboptimal participation rates threaten the cost-effectiveness of screening programs [7–9]. Despite aligning with the average uptake rate in Europe for BC screening (48.2%, ranging from 19.4% to 88.9%) [10], the current uptake rate in Flanders stands at 54.1% [11] %, slightly above the European average of 48.2% (ranging from 19.4% to 88.9%) but falling short of the official target of 75% [12].

### Breast cancer screening among underserved communities in Flanders

Individuals from underserved and minority communities frequently encounter additional barriers to accessing preventive health services, influenced by economic limitations, health literacy, language obstacles, and cultural beliefs [13–15].

In Flemish municipalities where incomes are below the poverty line, BC screening uptake is 15% lower than the average [16]. Municipal-level studies further reveal that socioeconomic status (SES) indicators such as crowded living conditions, high population density, and a significant proportion of foreign-born residents are associated with lower BC screening uptake [17, 18].

Qualitative insights from minority communities in Antwerp, the largest city in Flanders, highlight a deeper complexity in the issue. Focus group discussions (FGDs) conducted in 2012 with 20 ultra-orthodox Jewish women highlighted the importance of a supportive environment. Despite fears of pain and anxiety about screening results, all participants expressed high motivation for health behaviors and had participated in the breast cancer screening program previously. They emphasized the community's influence, where decisions made by one individual often influenced the others. Family support was a primary motivator, with participants stressing the importance of staying healthy and alive for their children. Cultural barriers were also noted, particularly regarding discomfort with male healthcare providers performing the examination [19].

On the other hand, FGDs conducted in 2014 with 17 first-generation Turkish women revealed that exposure of breasts to others was reportedly a concern for the husbands, negatively influencing participation. Family members served as key facilitators, assisting with tasks such as retrieving invitation letters, providing translations, accompanying participants to appointments, and clarifying screening results. Logistical issues such as lack of transportation were mentioned as significant barriers. Additionally, a general attitude of neglect towards health behaviors and preventive measures, as well as fear of hospitals, further impeded screening uptake [20].

Given that barriers can arise as early as the receipt of the invitation letter, promotional materials produced by the Centre for Cancer Detection (CvKO) in Flanders must be clear and tailored to the specific needs of target communities.

Research indicates that interventions such as appointment reminders can significantly enhance participation rates; for instance, randomized controlled trials (RCTs) have shown that reminders can improve participation among non-responders by up to 90% [21]. However, the BCSP in Flanders has not yet implemented reminder systems due to insufficient robust evidence regarding their effectiveness in the local context.

This study seeks to fill this gap by:

- Exploring and reporting factors influencing participation in the BCSP among women from underserved communities in Flanders.
- Developing an effective and accessible reminder letter, by integrating the perspectives of both the target audience and domain experts to encourage participation in the BCSP among women from underserved communities in Flanders.

## Methods

### Study setting

Since 2001, in Flanders, a BSCP in line with the European Guidelines for Quality Assurance [8] was established by the Flemish Government and implemented by the Centre for Cancer Detection (CvKO) [22]. Eligible women aged 50–69 are recruited for the program through a personalized invitation letter with a set time and location.

A mammography is provided every two years and is paid for by the health insurance system.

It should be noted that in Belgium, health insurance is part of the social security system. Everyone has to have health insurance and must join an accredited health insurance fund. However, those who do not want to or cannot afford to join a private health insurance fund can join the ‘Hulpkas voor Ziekte- en Invaliditeitsverzekering’ (Auxiliary Illness and Disability Insurance Fund) for free [23]. Still, people who are not legally residing in Belgium do not have access to this service and do not receive invitation letters for mammography screening.

The development of this reminder letter was undertaken as part of the ‘ENTER: Equity in Breast Cancer Screening in Flanders’ project, with the objective of pilot-testing its effectiveness in increasing BCSP participation among previous non-responders. In this ongoing trial – which will be discussed elsewhere – the control group will receive only the official invitation, while the intervention group will receive both the official invitation and the newly developed reminder letter.

### Study population

Underserved women were defined as individuals who face significant barriers to accessing essential services, including healthcare, due to socioeconomic disparities, cultural or linguistic challenges, and systemic inequity [24]. In this study, we did not directly assess whether participants self-identified as underserved; rather, we recruited them through organizations that specialize in supporting individuals considered part of this target group. These organizations included the open learning centers of Linkeroever and Luchtbal in Antwerp, the walk-in center in Mechelen, Saamo (Tackling Exclusion Together) in Ostend, and FMDO (Federation for Global and Democratic Organizations) in Ostend. It is important to note that we did not inquire about the legal status of foreign participants, allowing for the inclusion of individuals from various backgrounds. Characteristics of study participants are listed in Table 1.

**Table 1:** Characteristics of study participants (April-June 2023, Flanders, Belgium)

Group	Location	Participant	Native language	Country of origin	Age	Receipt Invitation letter	Mammogram	Notes
FGD1	Open learning centre – Linkeroever, Antwerp	Participant 1*	/	/	72	Yes	/	
		Participant 2	Polish	Poland	66	Yes	Yes	
		Participant 3*	Arabic	/	49	No	No	Anxious/scared, not attentive during the discussion.
		Participant 4*	Arabic	Jemen	39	No	No	
		Participant 5	Assyrian, Kurdish, Arabic	Syria	66	/	/	
		Participant 6	Spanish, Italian	Spain	61	Yes	Yes	
		Participant 7	Somali, Arabic	Somalia	58	Yes	/	
		Participant 8	/	/	65	Yes	Yes	
		Participant 9	/	/	52	/	/	
		Participant 10	/	/	55	/	/	
		Participant 11	Dari Persian, Pashto	Afghanistan	50	No	No	
		Participant 12	Dari Persian, Pashto	Afghanistan	65	/	/	
FGD2	Open learning center – Luchtbal, Antwerp	Participant 1	Arabic, French	/	50	No	No	
		Participant 2	Dari Persian, Pashto	Afghanistan	47	No	Yes	
		Participant 3	Amharic	Ethiopia	45	No	No	
		Participant 4*	Creole	/	33	No	No	
Individual interviews	Walk-in center – Mechelen	Participant 1*	Dutch	Belgium	72	Yes	Yes	
		Participant 2	Dutch	Belgium	52	Yes	Yes	Had a mammogram taken on the day of the interview.
		Participant 3	Dutch	Belgium	65	Yes	Yes	
Co-creation session	Vrouwenklap project FMDO -Ostend	Participant 1	Berber	Morocco	50	No	No	
		Participant 2	Dutch	Belgium	50	No	No	
		Participant 3	Berber	Morocco	53	Yes	Yes	
		Participant 4*	Arabic	Iraq	33	No	No	
		Participant 5*	Arabic	Syria	39	No	No	
		Participant 6	Ukrainian	Ukraine	59	/	Yes	
		Participant 7*	Arabic	Saudi Arabi	29	No	No	
		Participant 8*	Berber	Morocco	39	No	No	
		Participant 9*	Arabic	Syria	29	No	No	
		Participant 10*	Uzbek	Uzbekistan	74	/	/	
		Participant 11*	Armenic	Armenia	72	Yes	Yes	
		Participant 12	Arabic	Iraq	51	Yes	Yes	
		Participant 13	Dari Persian, Pashto	Afghanistan	58	Yes	Yes	
		Participant 14	Arabic	Syria	62	Yes	Yes	

* = Not in the target age group for BC screening (50–69)

/= Missing information

### Study design

This study follows a phenomenological qualitative design, focusing on understanding the lived experiences of underserved women in relation to the BCSP and its invitation letter. Phenomenological techniques were chosen to explore how participants perceive, interpret, and respond to the communication about the BCSP [25]. Thematic analysis was identified as an effective method for capturing key themes. Bracketing was utilized to minimize researcher biases, ensuring an open stance toward participants' perspectives [26]. Finally, constructivist inquiry supported the active construction of meaning from participants'experiences, facilitating the design of a reminder letter that resonates with participants’ insights and needs [27].

The co-creation was guided by principles embedded in MH Europe and Health Cascade initiatives [28, 29], however, this was only applied to the data collection process.

### Iterative process and timeline

The development of the tailored reminder letter was part of an iterative co-creation process, which included multiple stages of data collection and consultation.

According to the study objectives, the project comprised two key steps:

- Focus Group Discussions (FGDs) and interviews
  Two FGDs and three individual interviews were conducted between April and June 2023 with underserved women living in Flanders. During these meetings, participants were asked to provide personal information such as age, native language, area of residence, receipt of the screening invitation letter, and previous mammography. Participants were informed that they were not obliged to disclose personal details if they were uncomfortable doing so.
  FGDs were audio-recorded with participants'consent. Each recording was transcribed verbatim to support a detailed qualitative analysis.
  A topic list was developed and used consistently across all FGDs and interviews to ensure focus, though this list was adjusted after the first FGD based on emerging themes and insights. The FGDs concentrated on identifying the most common barriers and facilitators to participation in the BCSP and evaluating the official invitation letter and its possible iterations.
- Development of the tailored reminder letter
  Insights from the FGDs informed the creation of a tailored reminder letter. This process involved two main components:
  - ▪ Delphi consultations: We employed the Delphi method to achieve consensus among a panel of stakeholders, including the BCSP program manager and representatives from local organizations working with underserved women (including Solidaris, Logo, FMDO, AZ Damiaan, Saamo, and Community Health Workers). The reminder letter was refined through consultations with sector experts after each FGD. In total, three rounds of expert consultations were conducted to finalize the letter's content.
  - ▪ Co-creation session: In October 2023, a co-creation session with underserved women was organized in collaboration with Saamo and FMDO during a set event known as ‘Vrouwenklap’ (Women’s talk), a project for women with a migration background in Ostend that focuses on language and encounter [30]. Participants evaluated the revised letter during this session, providing feedback on its clarity and relevance. The final version of the reminder letter was sent to experts from the Delphi panel for a member check, ensuring that all feedback was incorporated and consensus was reached.

**Fig. 1:**
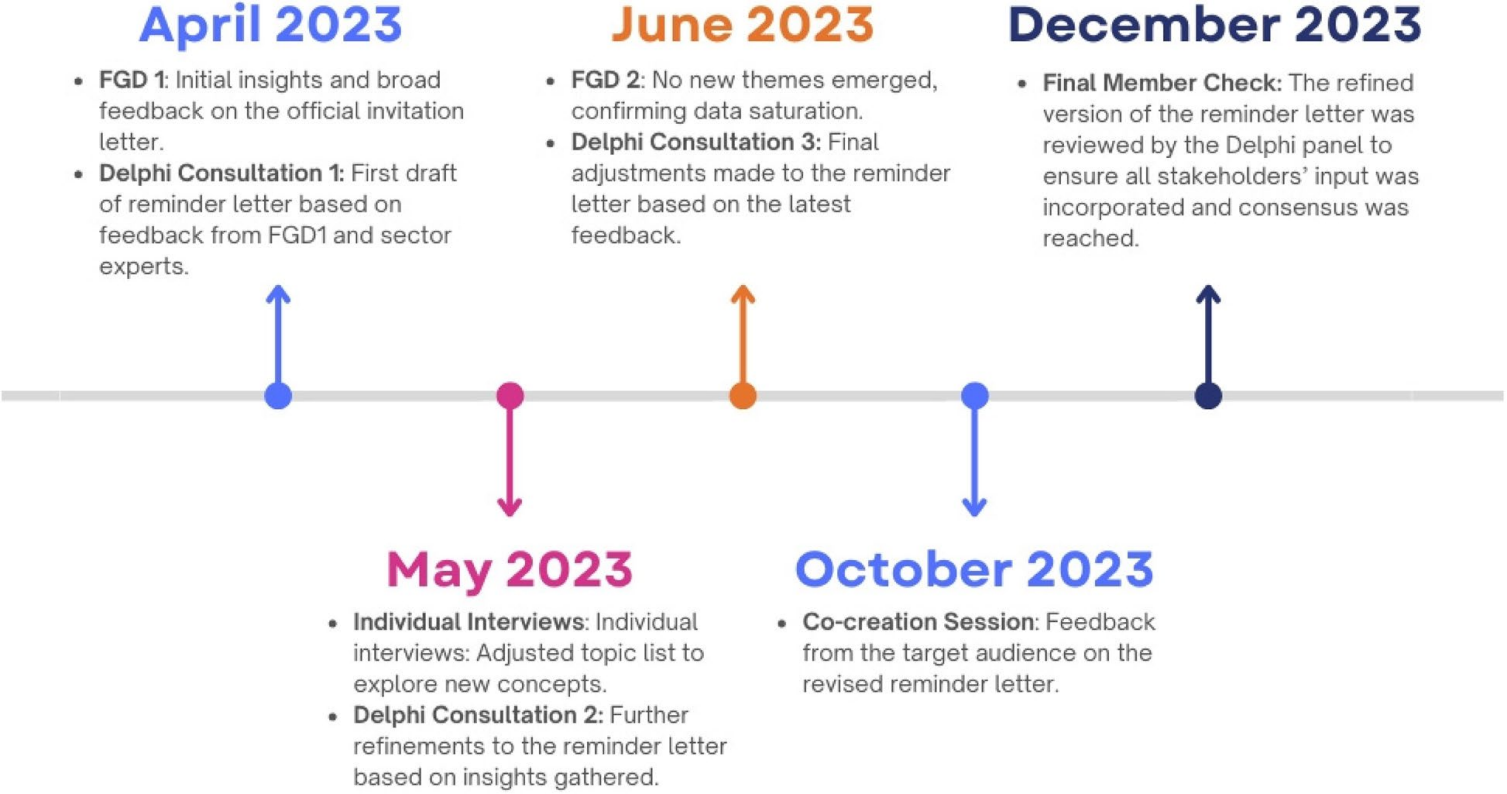
Timeline for the development of a tailored reminder letter for breast cancer screening (April-December 2023, Flanders, Belgium)

The process unfolded as shown in Fig. 1.

### Data saturation

Data saturation was achieved when no new insights emerged during the last FGD. After FGD1, which provided a broad spectrum of information, the topic list was refined. Individual interviews allowed for a deeper exploration of certain issues. FGD2 confirmed that the identified concepts were well-represented, marking the saturation point.

### Data analysis

Data from the FGDs and interviews were analyzed using a combination of qualitative thematic content analysis and visual methods. The NVivo software (version 14.23.0, Lumivero) facilitated systematic coding, categorization, and thematic analysis of the qualitative data.

The analysis process followed the Quagol method for qualitative research, involving the creation of initial codes based on the raw data, followed by successive rounds of coding, recoding, and synthesis to identify recurring themes and patterns [31].

In line with a constructivist perspective, our analysis acknowledged that meaning is co-created through the interactions between participants and researchers; as such, our iterative coding process involved regular team discussions and reflexive adjustments to ensure that emerging themes accurately reflected the diverse contexts and interpretations of the participants’ lived experiences.

**Fig. 2:**
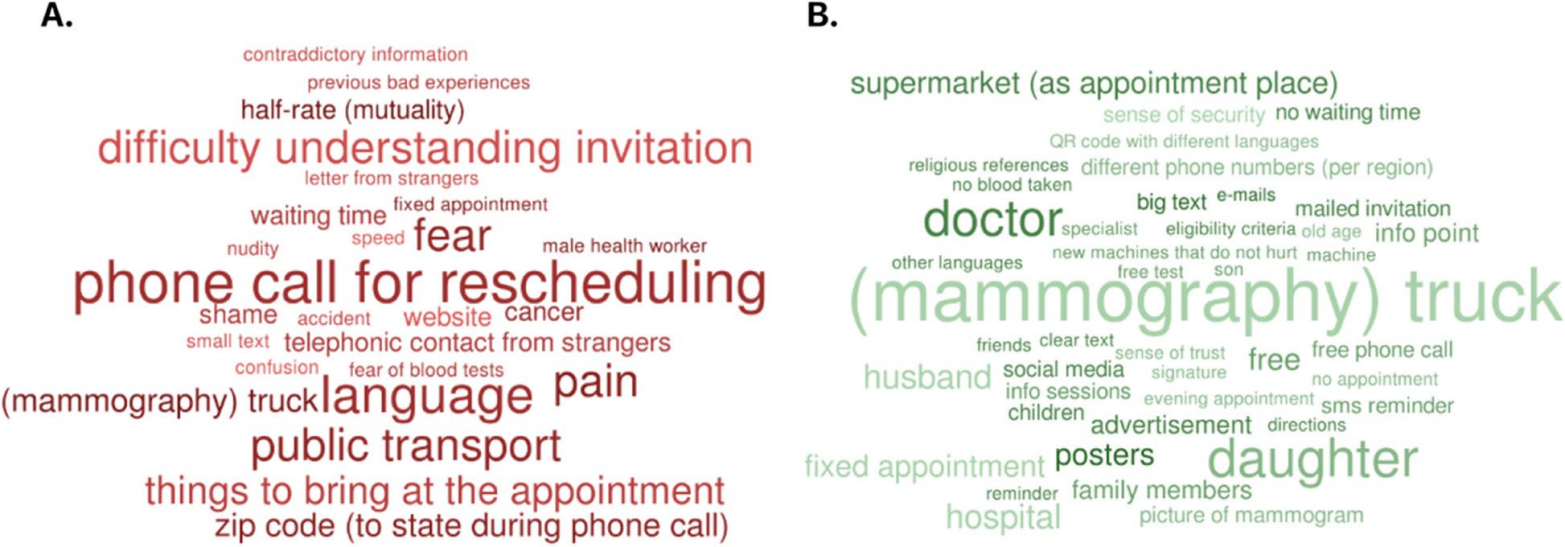
Barriers (**A**) and facilitators (**B**) to participation to the breast cancer screening program identified by study participants (April-June 2023, Flanders, Belgium)

To visually represent key themes, we generated word clouds (Figs. 2 and 4), where the frequency and importance of terms were illustrated through variations in font size and color. This approach helped quickly identify the most frequently mentioned concepts and concerns raised by participants.

## Results

Between April and October 2023, a total of 33 participants were interviewed. Their characteristics are shown in Table 1.

### Factors influencing participation in the breast cancer screening program

#### Preventive behavior

The concept of prevention (“preventie” in Dutch) was not easily understood by the participants. During FGD1 and 2, women from different countries including Somalia, Ethiopia Afghanistan and Poland shared their experiences with healthcare, highlighting that it is not customary or considered normal for them to visit a GP or undergo an examination solely for preventive purposes. During FGD1, a question was raised from the participants what they should do when they notice a lump in their breast or experience any concerning symptoms. It was observed that participants tended to downplay such symptoms and only sought medical attention when they perceived themselves to be truly unwell. This suggests a general tendency to visit the doctor primarily when experiencing sickness rather than engaging in proactive healthcare practices.

Even among Dutch-speaking participants, there were differences in understanding. One Dutch-speaking woman in an individual interview did not fully grasp the concept of prevention, stating that having a mammogram is "*something you just do*", indicating it is a routine task rather than a consciously chosen preventive measure. Conversely, the other two Dutch native speakers who were individually interviewed immediately understood the concept of prevention and had already participated in the screening program. A Spanish-speaking woman in FGD1 also understood the concept of prevention, drawing parallels with the Spanish word “prevenir".

#### Knowledge of BC

During both FGDs, participants asked questions and displayed general confusion about breast cancer. FGD1, in particular, revealed substantial gaps in the participants'understanding of breast cancer, its symptoms and risk factors. While participants were familiar with the concept of a lump, describing it as "*feeling* [the breast tissue] *harsh*", and some even physically mimicked the presence of a lump or pain in the breast, there was still a considerable lack of clarity.

First, there was notable confusion and misinformation regarding who can develop certain types of cancer. For example, some participants believed that colon cancer only affects men, and there was uncertainty about whether men can develop breast cancer. Participants raised questions about why individuals who do not engage in known risk behaviors, such as “*muslims, who do not drink alcohol*” still develop cancer.

Some participants also mistakenly believed that the examination involved taking a blood sample instead of a mammogram. A participant also confused the concept of biopsies and endoscopies in the context of breast cancer diagnosis.

#### Potential barriers to participate in the BCSP

Fear of experiencing pain and receiving a negative result were common barriers.

All women were asked why they were or were not participating (if applicable) or why some women would choose not to participate. During an individual interview, a participant believed that the fear of pain might be a reason why a lot of women do not participate. She heard about “*a new machine that is painless*” and suggested giving more information about that in order to reduce barriers to access. In this regard, one of the participants in the co-creation session stated:

“*I don't want to do it. If there is another type of test available, I would consider it. But I don't want to have my breast placed between a machine*” *(F 50y, Participant 1, Co-creation session).*

While fear of pain and discomfort were mentioned as common barriers, it should be noted that not everyone identified or experienced them as obstacles. During FGD1, a woman stated:

*“Yes it hurts, but it is important, so pain is not a problem” (F 61y, Participant 6, FGD1).*

Also during the co-creation session, women who participated in screening indicated that “*it is not painful, it depends on the person”* and also that “*it's a simple check, it is unpleasant but not painful”*.

With regards to the fear of receiving a negative result, a participant of FGD1 mentioned being scared the first time she received the letter, but:

*“When it was not the first time anymore, I had no more fear … I got a healthy result, and this helps to do the screening” (F 65y, Participant 8, FGD1).*

Having a personal connection with someone who has had or currently has breast cancer was perceived as both a facilitator and a barrier to participation.

 For instance, a participant said:

*“I think it's important because one of my nieces got breast cancer and so did four or five of my classmates from before” (F 65y, Participant 3, Individual interview).*

Some participants also suggested that telling more stories and experiences of women with BC might increase willingness to get screened. However, during FGD1, there was a participant who had experienced the loss of her sister due to breast cancer. She chose not to discuss this topic and also did not disclose her participation status in the screening program. This highlights how such narratives can evoke mixed reactions, as they may encourage some individuals to seek screening while also prompting emotional discomfort for others.

Many participants suggested that a general mistrust of the healthcare system or fear of the hospital environment might be additional barriers to participation. For example, a participant in an individual interview expressed frustration with the logistical challenges of healthcare, saying, “*when I have to call the hospital, I often have to wait in line for a long time*” *(F 72y, Participant 1, Individual interview).* Additionally, there was a complaint about the lack of general practitioners in the area, with one participant explaining that she would prefer to consult her GP rather than visit a hospital for a mammography. Despite these challenges, the Dutch word for hospital ("ziekenhuis") was familiar to most participants, indicating that the hospital remains a recognized place of referral.

Finally, transportation was highlighted as a significant barrier to accessing hospitals or other mammographic units, especially for those with reduced mobility or those who rely on others for mobility assistance. This issue was brought up multiple times by a woman *(F 72y, Participant 1, Individual Interview)* who emphasized her difficulties in getting to the screening location.

A crucial barrier appeared to be a lack of understanding or misconceptions about the screening process. Some participants mistakenly interpreted the invitation letter and the term "breast cancer" as an indication that they already had cancer, which heightened their fear and led to rejection of the program.

Additionally, some women had not yet received the letter and, when shown an example, did not understand its purpose without a thorough explanation.

#### The role of the support system

A notable pattern among respondents was their reliance on family or trusted individuals for support, highlighting the importance of involving close networks in the prevention process.

In general, most participants in both FGD’s sought help from family members, particularly their daughters, sons, and husbands, when they encountered difficulties in understanding the information.

When asked about changing an appointment (as described in the invitation letter), many participants indicated that they would rely on their daughters for help. A participant from FGD1 noted that her daughter, who is a nurse, would be the one to make the appointment for her. Similarly, a participant in FGD2 emphasized the advantages of having family members who understand the language, in contrast with her friends who might struggle:

“*My friends don't understand, but it's easy for me. When the letter arrives, my daughter understands, my husband understands” (F 50y, Participant 1, FGD 2)*.

In this regard, a participant suggested that educating younger generations about the screening program could serve as an additional informational channel, not only for their own awareness for future reference, but also to assist and support their mothers in navigating the healthcare system. Husbands also played a significant role in helping participants, but this support depended on their ability to understand and speak Dutch.

With regards to the social network, some participant explicitly verbalized that they would discuss BC screening with friends or members of the community (*F 61y, Participant 6; F 55y, Participant 10, FGD1*). However, this did not seem like a common experience. In particular, participants attending the walk-in center in Mechelen opted for individual interviews as they were unwilling to discuss the topic with peers. In FGD1, three women explicitly stated that they would refrain from discussing the topic with friends or members of their community. Notably, one participant nodded in disagreement, saying “*no, not talking*” (*F 65y, Participant 12, FGD1*) when asked if she would engage in discussions about BC screening. Despite this, participants in the group discussions did show a degree of openness when talking to peers in the session. Some expressed their ideas and perspectives, while others discussed challenges they faced in understanding the screening program.

Participants often sought advice or information from healthcare professionals, including their general practitioners (GPs). When language barriers existed, they mentioned once again that they would ask their relatives to assist with communication with the doctors. Alternatively, a commonly described approach was to simply hand over the letter to their GP for guidance. Additionally, some participants reported seeking advice from their local pharmacy when they needed further clarification.

Another important source of professional information was the individuals working or volunteering at the locations where the FGDs took place. During these meetings, a teacher or facilitator was present to provide assistance, and participants frequently relied on them to ask questions or seek help. Some participants even spontaneously mentioned the teacher as a go-to person for assistance when they did not understand something.

Although participants did not explicitly mention the importance of trusting professionals they sought assistance from, their behaviors suggested it was a significant factor. Trust was particularly crucial in the walk-in center environment (where the individual interviews took place), where participants reported preferring one-on-one conversations with trusted professionals while avoiding discussing their concerns in the group.

#### Accessibility and low threshold

Many participants emphasized the significance of having breast screening services conveniently located and easily accessible. Moreover, the recognizability of the screening facility was considered valuable by the participants.

During FGD1, participants spontaneously mentioned the "mammobile" (a mobile examination truck), widely recognized by all participants. The term "screening" was unfamiliar to the participants from this group, but when discussing the mammobile, the concept of screening and specifically the examination by mammography became clear. Thus, it appears that the mammobile provided a recognizable and tangible representation of the screening process.

During FGD2 only one participant was familiar with the concept “mammobile”. However, when participants in this group were asked about to express their thoughts on a mobile examination truck, the following arguments were provided:

“*Many people avoid going to clinics or hospitals, so having a mobile unit like a truck is a good idea. It makes it easier for them*” *(F 47y, Participant 2, FGD 2).*

One participant mistrusted the concept of the mammobile and would prefer “*the hospital* [because there you can get] *more professional care” (F 50y, participant 1, FGD 2).*

In the individual interviews, participant 2 heard of the mammobile through a colleague in Antwerp. She found the idea was “*very good, practical”.* For many participants, including those who did not hear of the mammobile before, this was perceived as a valuable system because it could potentially increase accessibility, especially for people with mobility issues.

It should be noted that when the concept of the mammobile was not known there were also some misinterpretations. For example, some participants in FGD1 mistakenly believed that the mammobile does not require an appointment. Overall, the presence of the mammobile enhanced the visibility and recognition of the screening program. The appointment location specified in the example letter was noticed and discussed by certain participants. During an individual interview, it was highlighted that reaching the specified location (the regional hospital) was not feasible for the participant. Additionally, one participant expressed a dislike for the location mentioned in the example letter due to a previous negative experience there. Interestingly, supermarkets or parking areas near supermarkets were frequently mentioned as desirable and recognizable locations for the mammobile’s stops.

#### Information channels

Upon gaining a better understanding of the BCSP, participants suggested various strategies to improve its visibility. Common recommendations included leveraging social media platforms like Facebook and placing posters in crowded public spaces. However, one participant expressed doubt, stating that these methods might not significantly influence individual behavior.

Communication through the BCSP website was not a preferred information channel. Participants in both FGD1 and FGD2 commented that the website was too complex to understand and would likely not be used. One participant also mentioned that she does not fully comprehend how to use the internet, highlighting challenges related to digital literacy within this group.

The potential use of SMS or WhatsApp reminders for the screening program was explored. One participant suggested that an SMS reminder could be effective, similar to those sent by GPs for appointments. However, concerns were raised about trust, as participants expressed a general mistrust of receiving texts or calls from unknown numbers. Additionally, language barriers were identified as another significant obstacle.

Throughout the FGDs and individual interviews, it became clear that many participants preferred to seek information and assistance from trusted individuals in familiar settings. Walk-in centers and schools offering Dutch language classes (which were the settings chosen for these interviews) were seen as comfortable and low-pressure environments where referral people could be approached. Doctors were also mentioned as trusted figures, leading to the suggestion that community health centers could serve as potential venues for hosting informational sessions. Notably, during FGD1, numerous questions were raised by participants, indicating a need for a comprehensive information hub that could address these queries and provide further guidance.

Figure 2 present word cloud visualizations illustrating the barriers and facilitators to participation to the BCSP in Flanders, as identified by interviewed women, based on the frequency of mentions.

#### Help-seeking behavior

Help-seeking behavior in the context of BC prevention refers to the actions and decisions individuals take to seek medical advice, information, or support regarding BC screening and preventive measures. This behavior can be influenced by various factors, including awareness of BC risks, knowledge of screening options, perceived vulnerability, cultural beliefs, access to healthcare, and social support networks. It entails various steps, including recognizing the need for assistance and actively searching for suitable resources or support systems [32]. In our FGDs, participants demonstrated increased help-seeking behavior after gaining a better understanding of the screening program. This included taking home informational materials and asking additional questions. Participants also showed help-seeking behavior by indicating that they would consult family members or a doctor for guidance. The overall perception of the program was favorable, with many acknowledging its importance and appreciating its availability to the population.

## Development of a reminder letter

### Experience and evaluation of the official invitation letter

Overall, participants share positive experiences with an invitation letter as an information channel and expressed a sense of trust of receiving the information in that way. One participant *(F 50y, Participant 1, FGD2)* specifically mentioned her appreciation for this type of communication, describing it as a "*better”* channel and expressing trust in its reliability. She further mentioned that while some individuals like herself may not fully understand the letter, her daughter and husband are able to comprehend its content. Another participant *(F 52y, Participant 2, Individual Interview)* also expressed satisfaction with the letter as an information channel but emphasized the need for additional reminders, also in the form of letters. She expressed concern that after a certain point, invitations are no longer sent by letter if someone does not participate, which she found unfortunate given the importance of the program. In reality, however, invitations only stop if an individual actively opts out. On the other hand, a few participants suggested that some might throw the letter away thinking it is an advertisement.

**Fig. 3:**
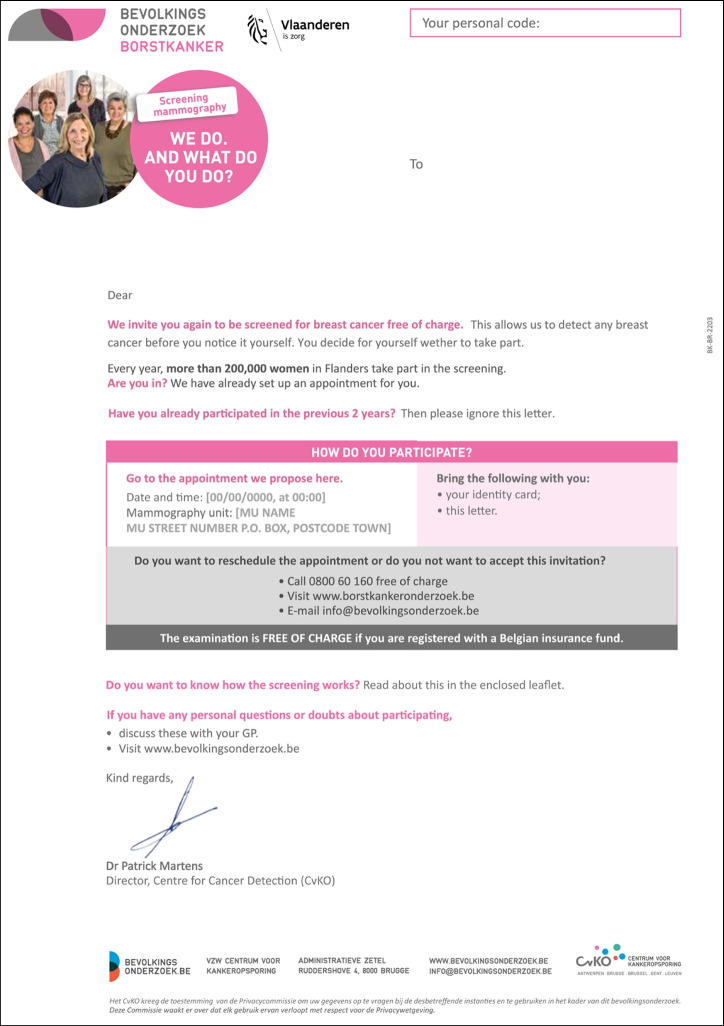
Official invitation letter for breast cancer screening adopted by the Centre for Cancer Detection (CvKO) in Flanders (English translation, 2023)

Participants highlighted that the most important details for immediate clarity included the appointment date, location, contact information, and the fact that participation is free of charge. Although this information was present in the original letter (Fig. 3), it was obscured by distracting elements, complex sentence structures, and grammar that diverted attention from the key points.

To assess comprehension, respondents were asked to read sentences from the letter aloud and explain what they understood. During the discussions, suggestions were made to transform this information into a more functional format that would enhance knowledge, provide clear instructions, and support decision-making.

#### Text structure and visual aspect

In FGD1, a participant described the letter as excessively lengthy and complex. The use of advanced vocabulary and grammar beyond an A2 level was a barrier for many. With the exception of native Dutch speakers, most participants struggled to comprehend the full text and only relied on a few familiar terms to understand the letter’s purpose. Visually, the letter was described as being too busy, and the font was noted as too small by multiple participants.

A description of the main issues highlighted by participants are presented below:

**Table:** 

Dutch	English translation	Issue
		Participants could not immediately link the group of women in the picture with the BCSP. The setting did not recall a mammographic unit, as it was not clear if women were indoor or outdoor. It was also noted that a more diverse group could be representative of a broader audience.
De screeningsmammografie	Screening mammography	Certain terms including screening and mammography were not immediately understood and required thorough explanation. Only a few participants during the co-creation session and FGD 1 knew the word “mammography”.
Wij doen het. En wat doe jij?	We do it. And what do you do?	The slogan was not understood by participants during the co-creation, who did not link this sentence to the BCSP. A participant said: "*I don't understand. We're doing it, but what are we doing?*" *(F 39y, Participant 5, Co-creation session).* Another said: "*What is this?".*
(Dankzij borstkankerscreening) kunnen we een mogelijke borstkanker vinden voordat je er zelf iets van merkt	(Thanks to breast cancer screening) we can find a possible breast cancer before you even notice it yourself	In FGD2, none of the participants were able to accurately state what was the meaning of the sentence.
Elk jaar doen meer dan 200.000 vrouwen in Vlaanderen mee aan het onderzoek	Every year, more than 200,000 women in Flanders take part in the screening	This was understood in FGD 2 as: "200 women in Flanders participate in research” *(F 33y, Participant 4, FGD2).*

#### Functionality

Participants reported feeling uncertain about the steps needed to reschedule their mammography appointment or seek further information, largely due to the confusing layout of the letter

Specifically, the letter included a box with practical appointment details, followed by additional text that covered similar content but provided a different set of instructions (Fig. 3). This disjointed presentation left participants unsure which guidance to follow, highlighting the need for a more cohesive format.

Participants also expressed confusion about the two separate links included in the letter: one directing to the BCSP page (< www.borstkankeronderzoek.be >) and another to the CvKO website (< www.bevolkingsonderzoek.be >), which covers all screening programs offered in Flanders. Having to navigate multiple webpages—and often needing to manually enter the URLs on smartphones or computers—posed a significant barrier.

Although the link to the BCSP website is presented under the prompt, “Do you want to reschedule the appointment?”, the letter does not explicitly guide users to the contact form necessary for changing their appointment. Moreover, although these websites offer information in multiple languages, this feature was not highlighted in the letter. As a result, participants experienced frustration and confusion when attempting to locate and use these resources, suggesting the need for more streamlined, user-friendly communication.

Finally, the letter lacked crucial details such as the call center's operating hours, leading to frustration among those who had previously attempted to call but received no response.

**Fig. 4:**
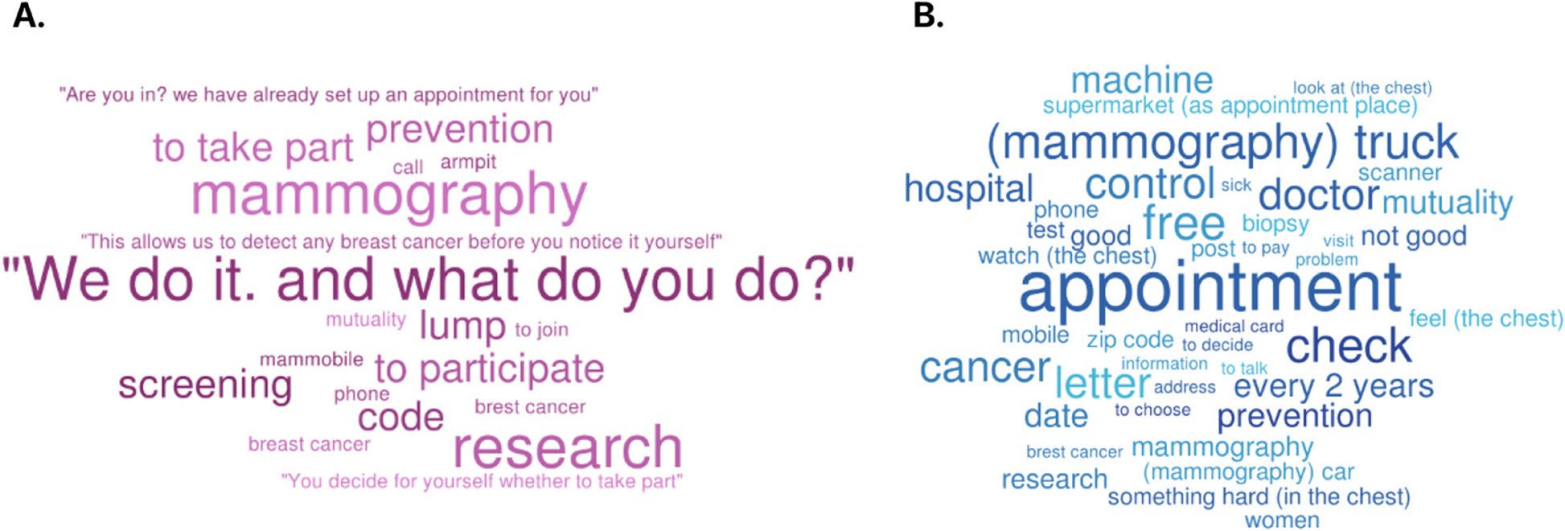
Words and expressions identified as difficult and complex (**A**) or (**B**) easy and accessible by study participants (April-June 2023, Flanders, Belgium)

Figure 4 present word cloud visualizations illustrating words and expressions difficult and easy to understand, respectively, as identified by interviewed women, based on the frequency of mentions.

### Drafting the tailored reminder letter

Following the first FGD, a draft of the reminder letter was created in collaboration with domain experts. This draft was then revised based on feedback received from participants during each subsequent FGD. This iterative process continued until an “interim” version of the reminder letter was completed (Supplementary Materials—Figures S3 and S4) and presented during a co-creation session in October 2023 with 14 participants.

Key updates in the “interim” version included:

- The introduction of a new logo, featuring a mammogram, along with a new slogan.
- The use of simplified language, incorporating key terms easily recognized by FGDs’ participants.
- Translation of the letter into 12 languages: Albanian, Arabic, Chinese, German, English, Farsi, French, Italian, Romanian, Russian, Spanish, Turkish.
- Explicitly listing the available languages and adding a QR code that links to a page where the letter can be downloaded in different languages.
- The use of a simplified list of instructions, featuring graphics, on what to bring to the appointment, where to find additional information, and how to change the appointment (including the call center's opening hours).
- The addition of an "attention-drawing" arrow to emphasize the importance of the letter and prevent it from being discarded.

#### The logo

The mammogram icon immediately stood out to the participants. At first glance, the chosen logo was clear and effectively communicated the purpose of the letter. Participants spontaneously recognized that it was related to a breast check-up, providing an immediate indication of the letter's content. One participant remarked, "*the picture of breasts is the first thing that strikes me" (F 50y, Participant 1, Co-creation)* while another asked: "*That's a mammography machine?" (F 59y, Participant 6, Co-creation).* Other comments included: "*The picture is clear. You immediately see what it is about*" *(F 53y, Participant 3, Co-creation)*, and *"it is a letter about the breasts, a clear image".* However, some participants associated the image with discomfort, with one saying: "*There is the breast in between. I don't like squeezing* [my breasts] *in the machine” (F 50y, Participant 2, Co-creation)*.

Despite the potential barrier posed by fear of the mammographic exam, the feedback suggests that the chosen pictogram is effective and clearly represents the subject matter. This clarity could have potentially allowed for avoiding the term "screening mammography" in the slogan, which is not widely understood by many.

It should be noted that various logo alternatives were explored, including other visuals for a mammogram and the use of pink ribbons. However, the pink ribbon did not convey the same clear message about breast cancer screening and mammography, as it was interpreted as "*a scarf*", "*an emblem to recognize a sick man or sick woman*" and even *"a tie*".

In a subsequent session with the expert panel, more realistic images of a mammogram were excluded, as they were considered potentially disrespectful to certain cultures.

#### The slogan

Including the term “screening mammography” in the logo led to confusion among participants. Some associated the term with the mammogram icon, while others did not understand it. A more familiar and easily understood term was “[health] check” (in Dutch “controle”), which proved to be a better fit for a generic slogan. Therefore, a phrase like *“Get checked for breast cancer for free”* was deemed more effective, as it avoided the term “mammography” and ensured clarity.

The use of the terms “cancer” (“kanker”) or “breast cancer” (“borstkanker”) was also evaluated. Given the immediate association with the icon, participants already understood that the focus was on breast cancer rather than another type of cancer. Therefore, excluding the word “breast” did not compromise clarity.

At the beginning of the session, one participant noticed the word "free". However, as the session progressed, it became clear that although the word appeared three times in the text, many participants did not notice it.

#### The "attention-drawing" arrow

Participants suggested adding a “warning” sign to indicate that the letter was important and should not be discarded as advertising. Two sentences were evaluated: *“Don’t throw this letter away!”* and *“This letter is important.”* Some participants found the first sentence “redundant” and even counterproductive, expressing a desire to discard the letter more upon seeing it. For this reason, “*This letter is important”* emerged as the better option, with a focus on the letter's significance rather than on instructing recipients on what to do. This phrase was placed in an attention-drawing arrow at the top right corner of the letter.

#### Multilingual options

A box listing the languages in which the translated letter could be downloaded via a QR code was added to the end of the text. Two design options were tested: one featuring circular flags representing the regions in which certain languages are spoken, and another listing the names of the languages written in their native script. The first option caused confusion because not all countries were represented by their flags, leading people to assume their language was not available if their country's flag was missing. For example, a Moroccan French speaker could see the French flag but not the Moroccan flag and mistakenly believed the letter was not available in French: *“The flags… countries are difficult for me. I don't know which language it is…" (F 38y, Participant 8, Co-creation*). Consequently, indicating the language names in their native scripts emerged as the better option.

During the co-creation session, two participants did not have smartphones. However, most participants successfully used the QR code to read the letter in their preferred language. Participants specifically recommended placing this section at the top of the letter. This way, it immediately catches the eye, allowing recipients to scan the QR code right away without having to read through the entire letter first.

**Table:** 

**Take-home messages**	
Despite substantial improvements, several important issues remained with the “interim” version of the letter:	
• Despite efforts to simplify and organize the content, the text still felt too busy. • The font size is too small, and important words (e.g., “free”) were not highlighted enough. • Certain words were still complex and not easily understood by the target audience; for example, using "huisarts" instead of "huisdokter" to refer to the family doctor, and "ziekenfonds" instead of "mutualiteit" to refer to the health insurance fund. • Efforts were made to simplify the phrase "[Thanks to breast cancer screening] *we can find a possible breast cancer before you even notice it yourself”* to *"*[an health check]* helps spot breast cancer early, before you feel anything in your breast".* However, this revision still lacked clarity. The concept of prevention was not always well understood by the target group, so the idea of detecting a lump early is not recognized as a desirable outcome. Furthermore, the term "feeling" ("voelen") in the context of detecting something in the breast was not fully grasped. Although "pain" would be more comprehensible, it does not apply to all cancer symptoms, and finding a suitable generic term remained challenging.	

### Finalizing the tailored reminder letter

**Fig. 5:**
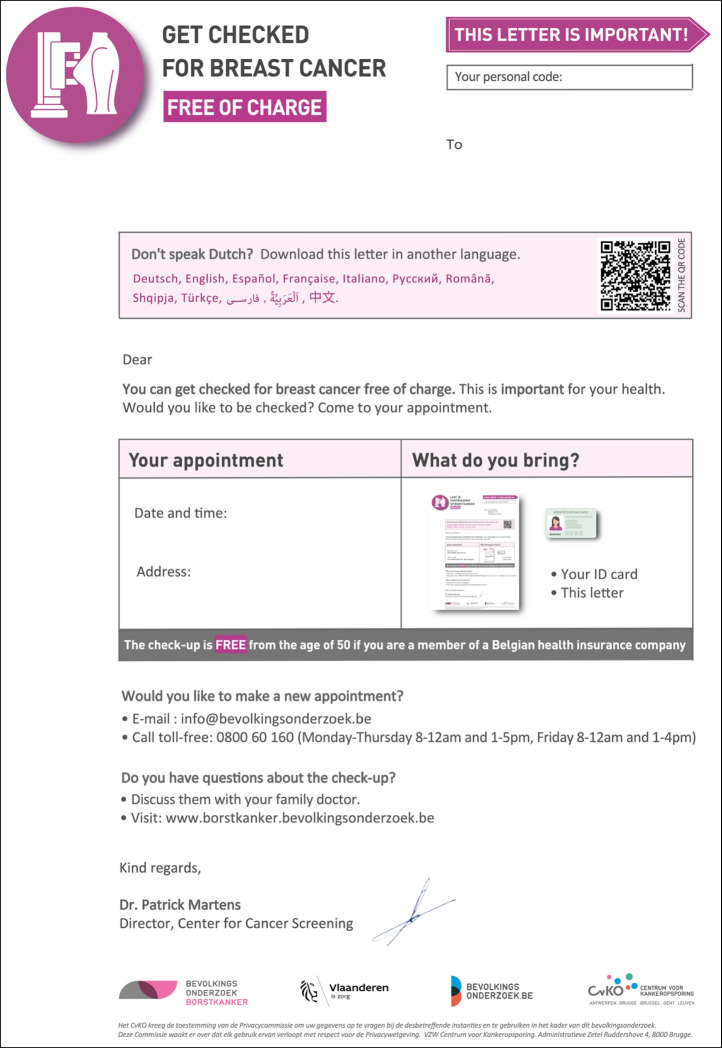
Reminder letter for the breast cancer screening program, tailored to the needs of underserved women living in Flanders (English translation, 2023)

Based on the feedback from the co-creation session, a final member check was performed with the expert panel, whose feedback was incorporated into the final version of the reminder letter (Fig. 5).

In particular:

- The new logo featuring a mammogram was permanently included, while the original text *"Screening mammography. We do. And what do you do?"* was replaced with the updated slogan *"Get checked for breast cancer free of charge" *("Laat je gratis controleren op borstkanker").
- An "attention-drawing" arrow was added at the top right, with the text *"This letter is important!"* ("Deze brief is belangrijk!"), to emphasize the letter's significance.
- The box listing available languages for translation (via QR code) was repositioned at the top of the letter for immediate visibility. Languages were displayed in their native script and highlighted in a different color to enhance readability.
- The phrase "[Thanks to breast cancer screening] *we can detect any breast cancer before you notice it yourself"* was removed and replaced with the simpler statement: *"This is important for your health"* (“Dit is belangrijk voor je gezondheid”).
- The box containing information about the appointment retained only essential details, with larger font size and visual support. The box highlighted that the check was free for women over 50 who were registered with a Belgian health insurance fund ("mutualiteit"). Information on how to reschedule the appointment was provided separately in the text.
- For rescheduling appointments, an email address and a free phone number with call center hours were provided. Questions about the screening were directed to one's GP (“huisdokter”) or the BCSP website < www.borstkanker.bevolkingsonderzoek.be >.
- The font size was increased, and bold text along with different colors were used to emphasize key concepts. The color palette shifted slightly from the original pink, which was sometimes associated with danger or pain (similar to red), to a more violet hue, still maintaining a color associated with breast cancer.
- The letter retained the signature of the CvKO director and relevant logos of the official invitation.

## Discussion

Being the third qualitative study to examine the experiences and needs of underserved populations in Flanders with regard to the BCSP, this contributes to a field with limited data, highlighting the unique challenges and opportunities for effective communication with these groups.

Effective communication is a cornerstone of public health initiatives, particularly for preventive health behaviors like cancer screening, which rely on the willingness and ability of individuals to engage with the services offered. Health behavior theories, such as the Health Belief Model, underscore the importance of perceived susceptibility, severity, benefits, and barriers. If communication about these programs is unclear or inaccessible, perceived barriers can overshadow the perceived benefits, resulting in lower participation rates [33].

A significant barrier to engaging in preventive health behaviors, including the BCSP, is a lack of familiarity with the concept of prevention [34]. Many participants, particularly those from cultures where medical visits are predominantly illness-driven, struggled to grasp the purpose of preventive screening. In these contexts, healthcare is often sought reactively, primarily when symptoms are present, rather than proactively for the preservation of health. However, once participants were educated about the BCSP, there was a noticeable shift in their health-seeking behavior. Participants began to appreciate the value of screening as a proactive measure to detect potential issues early, rather than waiting for symptoms to develop. This newfound understanding also helped to overshadow common fears related to pain or receiving a bad result.

These findings align with established frameworks in health communication. The EAST (Easy, Attractive, Social, and Timely) framework [35] emphasizes that clear calls to action, simple instructions, and limited options facilitate decision-making. Improving the design and accessibility of communication materials, such as the invitation letter, is also vital to ensure that all individuals, regardless of their linguistic or cultural background, can fully comprehend and engage with the BCSP. A recent systematic review on cultural appropriateness in health communication [36] highlights the importance of prioritizing cultural identity to acknowledge the diverse dynamics within racial and ethnic groups and to guide adaptive efforts for more effective messaging. This aligns with our findings, which underscore the necessity of culturally sensitive communication to effectively convey the importance of preventive care, as its understanding is highly influenced by cultural background.

Our experience indicates that community organizations working closely with underserved populations play a crucial role in facilitating communication on these themes across diverse groups. Additionally, partnering with language learning centers has proven effective in bridging language barriers and fostering trust.

While specific studies on partnerships between language learning centers and preventive health research is limited, broader evidence supports the effectiveness of community-based recruitment strategies in improving engagement and trust. Studies highlight that referrals from trusted community organizations significantly enhance recruitment success and participation rates, particularly when intermediaries such as educators and community leaders are involved in refining communication and outreach efforts- [37–39]. Additionally, research has shown that participants value not only ethical principles such as confidentiality and respect, and fair compensation but also seek reassurance that research will lead to meaningful impact [40]. Therefore, co-creation approaches involving these intermediaries are well suited, as they not only strengthen the methodology but also enhance participant motivation by allowing individuals to actively contribute to the research process.

However, research also highlights potential barriers to such collaborations, including limited staff capacity and time constraints within community organizations, which may reduce their ability to fully support research efforts. Despite these challenges, integrating language learning centers into preventive health outreach remains a promising and underexplored avenue that could strengthen engagement, improve comprehension, and foster long-term trust.

Participants in this study identified several issues with the official invitation letter, including its linguistic complexity, dense text, and visually cluttered design. The use of advanced vocabulary and grammar beyond an A2 level created significant comprehension challenges, leaving many participants reliant on familiar but insufficient terms to understand the letter’s purpose. Furthermore, the visual aspects of the letter, including an overload of information and a distracting logo, further impeded comprehension. The letter’s organization did not effectively highlight its most critical information, such as the appointment details, location, and the free nature of the screening.

In response to these findings, a tailored reminder letter was developed and iteratively refined based on the target group’s feedback. The revised letter aimed to address the identified barriers by simplifying the language, improving the visual layout, and making critical information more accessible. Key changes included the introduction of a more recognizable and clear logo, a simplified slogan, and translations of the letter into multiple languages, with this option highlighted using a QR code.

Despite these enhancements, some challenges remained. Although the letter was simplified, it still proved dense for certain readers. Additionally, the decision to use the term “[health] check” (“controle”) instead of “mammography” for clarity purposes was criticized by some members of the expert panel for who argued it lacked accuracy. These persistent issues underscore the challenge of balancing the requirement for comprehensive and detailed information with the need for clear and accessible communication for all literacy levels.

In addition, this study faced some methodological limitations. For instance, FGDs varied in size, with some being larger and others smaller, depending on participant availability and willingness to engage. While the varying group sizes allowed for diverse perspectives, they also presented challenges related to ensuring that all voices were heard, particularly in larger groups where dominant participants could overshadow quieter ones. This variation in group composition may have influenced the quality and depth of the discussions.

This risk was somewhat mitigated by the supportive atmosphere, which encouraged participation and helped quieter members speak up. However, the shared sense of vulnerability and trust could have introduced bias, with participants potentially conforming to group norms or providing socially desirable responses.

Furthermore, in certain cases, participant contributions could not be attributed to a single ID due to overlapping group discussions or non‐verbal expressions (e.g., gestures, mimicking), limiting the extent to which we could contextualize these excerpts with individual demographic data.

The potential influence of researchers and facilitators in shaping these dynamics warrants thoughtful consideration. This is especially essential when working with vulnerable populations, as personal empathy may unintentionally influence the interpretation of results. In this project, however, the researchers actively embraced reflexivity—deliberately examining their own mental frameworks and biases while incorporating rigorous evaluations by external analysts. This commitment to reflexivity became a guiding principle of this study, ensuring a more accurate interpretation of the interview data. Finally, it should be noted that while the co-creation was guided by principles embedded in MH Europe and Health Cascade initiatives [28, 29], this approach was applied primarily during the data collection process. We recognize that effective co-creation ideally involves stakeholders—especially end-users—at every stage of the project, from defining the problems, designing solutions, and evaluating outcomes. However, our decision to focus co-creation efforts on data collection stemmed from practical challenges related to working with a hard-to-reach population facing literacy barriers, which limited the feasibility of a fully co-created process. Future research efforts should strive for more comprehensive co-creation, ensuring that the voices and perspectives of end-users are integrated at each stage.

### Real-world application

This study is part of the project ‘ENTER: Equity in breast cancer screening in Flanders'. In line with its overarching goal of addressing disparities in BC screening participation among women with low-SES in Flanders (Belgium), the revised reminder letter is currently undergoing pilot testing in a RCT. Early, unpublished results from the RCT conducted by Ferrari et al. (2024), which compares the official invitation plus the new reminder letter to the official invitation alone among 7922 previous non-responders (where the expected participation rate is around 10%), show 80% relative increase in participation. Further steps are being taken to validate these findings within the subgroup of low-SES participants, which is expected to account for one fifth of the study population.

## Conclusions

The challenges identified in this study are not unique to the BCSP in Flanders but are reflective of broader issues in public health communication. Underserved communities often face multiple barriers to accessing health services, including language and literacy challenges, cultural barriers and economic difficulties.

Addressing these barriers effectively demands a culturally sensitive and collaborative approach. The iterative process used in this study, involving the direct engagement of the target population through focus groups and co-creation sessions, proved to be an effective method for refining communication materials. This approach not only identified and addressed specific issues with the existing materials but also ensured that the final product met the needs and preferences of the target audience.

Simplifying vocabulary, grouping related information, and providing direct links and language options improved the clarity and accessibility of the reminder letter, thereby fostering help‐seeking behaviors related to breast cancer screening.

## Supplementary Information

Supplementary Material 1.

## Data Availability

No datasets were generated or analysed during the current study.
