# Supplementary material for: From barriers to participation: co-creating an effective reminder letter for breast cancer screening among underserved women in Flanders
Source: Arch Public Health. 2025 May 14;83:132. doi: 10.1186/s13690-025-01591-7 (PMC12077022; doi:10.1186/s13690-025-01591-7)
Supplement: Supplementary file 1 — Supplementary Material 1. [file 13690_2025_1591_MOESM1_ESM.pdf]

From barriers to participation: co-creating an effective reminder letter for breast cancer screening among underserved women in Flanders - Supplementary Materials

Allegra Ferrari* (corresponding author: allegra.ferrari@uantwerpen.be) 1,2, Liesbet Van Bos* 3, Sarah Talboom 4, Wessel van de Veerdonk 1,3, Wendy D'haenens 3, Marina Pak 5, Marlies Descan 6, Stephanie Parmentier 6, Louise Van Collie 7, Pascale Sibiet 8, Mathieu Goossens 9, Guido Van Hal 1,9.

*Co-first authorship. Affiliations: 1 Social Epidemiology and Health Policy (SEHPO), University of Antwerp, Antwerp, Belgium; 2 Department of Health Sciences, University of Genoa, Genoa, Italy; 3 Centre of Expertise - Care and Well-being, Thomas More University of Applied Sciences, Mechelen, Belgium; 4 Centre of Expertise - Sustainable Business and Digital Innovation, Thomas More University of Applied Sciences, Mechelen, Belgium; 5 Federatie van Mondiale & Democratische Organisaties (FMDO), Ostend, Belgium; 6 ENTER project Working Group; 7 Lokaal Gezondheidsoverleg (Logo) Logo Brugge-Oostende vzw, Bruges, Belgium; 8 Stad Oostende, Ostend, Belgium; 9 Center for Cancer Detection (CvKO), Bruges, Belgium

The official invitation letter to the breast cancer sreening program is the property of the Centre for Cancer Detection (CvKO), Bruges, Belgium. The reminder letters and their iterations are the property of the researchers. These materials may not be used, reproduced, or distributed without written permission from the respective copyright holders.

Picture S1 – Official invitation letter for breast cancer screening adopted by the Centre for Cancer Detection (CvKO) in Flanders (Dutch version, 2023)

BEVOLKINGS
ONDERZOEK
BORSTKANKER

Vlaanderen
is zorg

Je persoonlijke code:

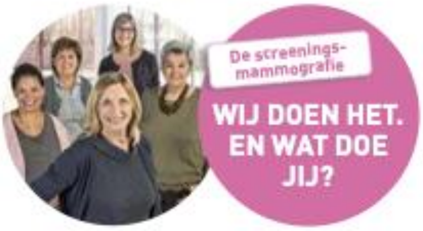

Aan

Beste

Je kan je om de twee jaar gratis laten onderzoeken op borstkanker. Zo kunnen we een mogelijke borstkanker vinden voordat je er zelf iets van merkt. Je beslist zelf of je meedoet.

Elk jaar doen **meer dan 200.000 vrouwen** in Vlaanderen mee aan het onderzoek.
Doe jij ook mee? Wij hebben al een afspraak voor je gemaakt.

HOE NEEM JE DEEL?	
**Ga naar de afspraak die we hier voorstellen.**<br>Datum en uur:<br>Mammografische eenheid:	**Neem het volgende mee:**<br>• je identiteitskaart;<br>• deze brief.
**Wil je de afspraak verplaatsen of wil je niet ingaan op deze uitnodiging?**<br>• Bel gratis naar 0800 60 160<br>• Surf naar www.borstkankeronderzoek.be<br>• Mail naar info@bevolkingsonderzoek.be	
**Het onderzoek is GRATIS als je aangesloten bent bij een Belgisch ziekenfonds**	

Wil je weten hoe het onderzoek verloopt? Lees het in de bijgevoegde folder.

Heb je persoonlijke vragen of twijfel je om deel te nemen?

- Bespreek het met je huisarts.
- Surf naar www.bevolkingsonderzoek.be

Met vriendelijke groeten,

Dr. Patrick Martens
Directeur Centrum voor Kankeropsporing

BEVOLKINGS ONDERZOEK.BE | VZW CENTRUM VOOR KANKEROPSPORING | ADMINISTRATIEVE ZETEL RUDDERSHOVE 4, 8000 BRUGGE | WWW.BEVOLKINGSONDERZOEK.BE INFO@BEVOLKINGSONDERZOEK.BE | CvKO CENTRUM VOOR KANKEROPSPORING

Het CvKO kreeg de toestemming van de Privacycommissie om uw gegevens op te vragen bij de desbetreffende instanties en te gebruiken in het kader van dit bevolkingsonderzoek. Deze Commissie waakt er over dat elk gebruik ervan verloopt met respect voor de Privacywetgeving.

Picture S2 – Official invitation letter for breast cancer screening adopted by the Centre for Cancer Detection (CvKO) in Flanders (English translation, 2023)

BEVOLKINGS ONDERZOEK BORSTKANKER

Vlaanderen is zorg

Your personal code:

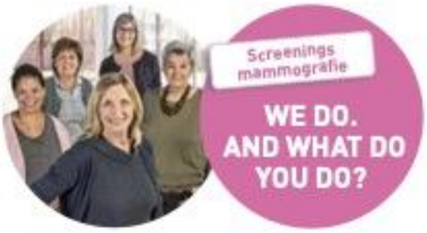

To

Dear

Every two years, you can be screened for breast cancer free of charge. This allows us to detect any breast cancer before you notice it yourself. You decide for yourself whether to take part.

Every year, **more than 200,000 women** in Flanders take part in the screening.
Are you in? We have already set up an appointment for you.

HOW DO YOU PARTICIPATE?	
**Go to the appointment we propose here.**<br>Date and time:<br>Mammography unit:	**Bring the following with you:**<br>• your identity card;<br>• this letter.

Do you want to reschedule the appointment or do you not want to accept this invitation?

- Call 0800 60 160 free of charge
- Visit www.borstkankeronderzoek.be
- E-mail info@bevolkingsonderzoek.be

The examination is FREE OF CHARGE if you are registered with a Belgian insurance fund.

Do you want to know how the screening works? Read about this in the enclosed leaflet.

If you have any personal questions or doubts about participating,

- discuss these with your GP.
- Visit www.bevolkingsonderzoek.be

Kind regards,

Dr Patrick Martens
Director, Centre for Cancer Detection (CvKO)

BEVOLKINGSONDERZOEK.BE | VZW CENTRUM VOOR KANKEROPSPORING | ADMINISTRATIEVE ZETEL RUDDERSHOVE 4, 8000 BRUGGE | WWW.BEVOLKINGSONDERZOEK.BE INFO@BEVOLKINGSONDERZOEK.BE | CvKO CENTRUM VOOR KANKEROPSPORING

Het CvKO kreeg de toestemming van de Privacycommissie om uw gegevens op te vragen bij de desbetreffende instanties en te gebruiken in het kader van dit bevolkingsonderzoek. Deze Commissie waakt er over dat elk gebruik ervan verloopt met respect voor de Privacywetgeving.

Picture S3 – Interim draft of a reminder letter for the breast cancer screening program, tailored to the needs of underserved women living in Flanders (Dutch version, 2023)

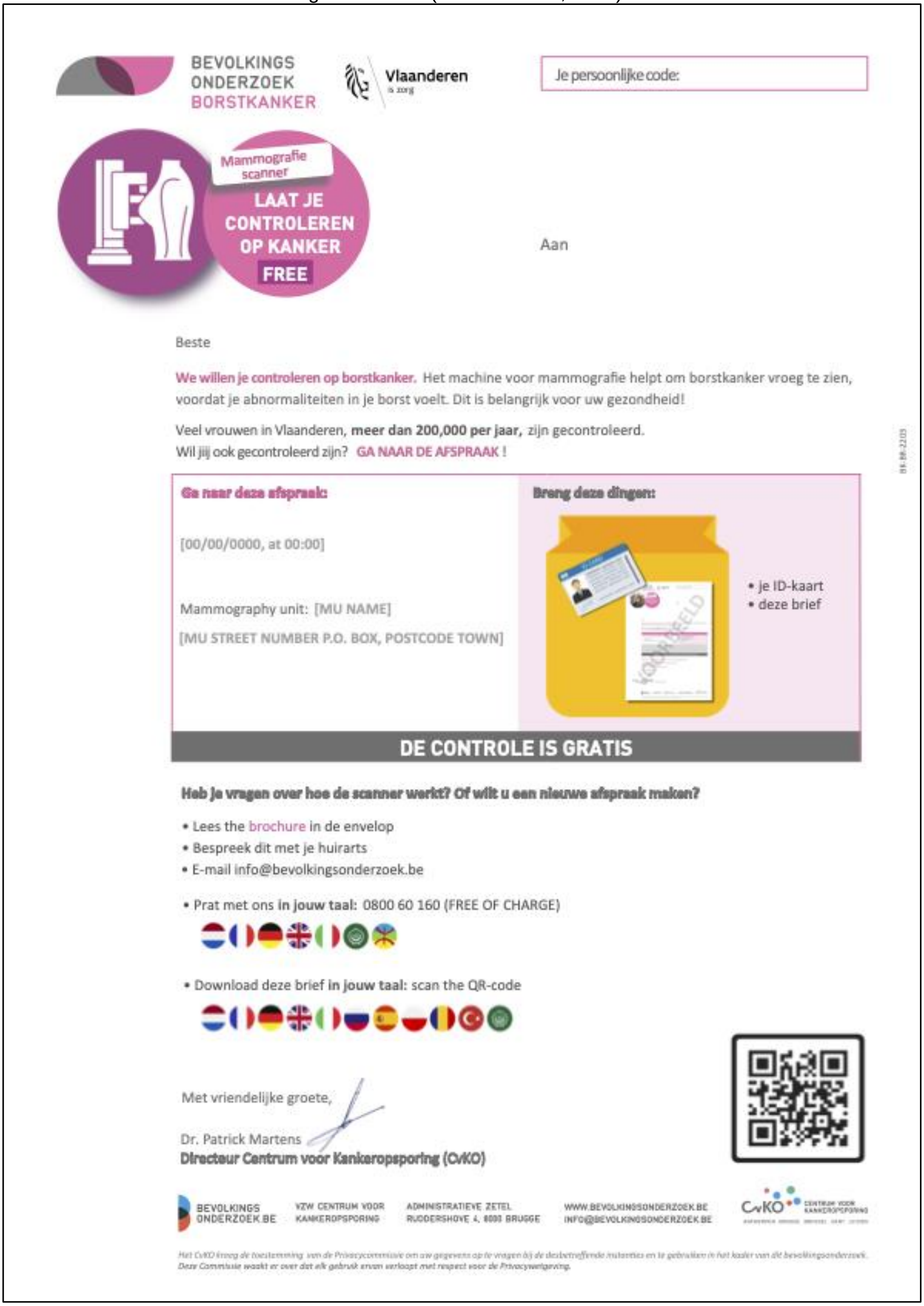

BEVOLKINGS
ONDERZOEK
BORSTKANKER

Vlaanderen
is zorg

Je persoonlijke code:

Mammografie scanner

LAAT JE CONTROLEREN OP KANKER

FREE

Aan

Beste

We willen je controleren op borstkanker. Het machine voor mammografie helpt om borstkanker vroeg te zien, voordat je abnormaliteiten in je borst voelt. Dit is belangrijk voor uw gezondheid!

Veel vrouwen in Vlaanderen, **meer dan 200,000 per jaar,** zijn gecontroleerd.
Wil jij ook gecontroleerd zijn? GA NAAR DE AFSPRAAK !

Ga naar deze afspraak:

[00/00/0000, at 00:00]

Mammography unit: [MU NAME]

[MU STREET NUMBER P.O. BOX, POSTCODE TOWN]

Breng deze dingen:

- je ID-kaart
- deze brief

DE CONTROLE IS GRATIS

Heb je vragen over hoe de scanner werkt? Of wilt u een nieuwe afspraak maken?

- Lees the brochure in de envelop
- Bespreek dit met je huiarts
- E-mail info@bevolkingsonderzoek.be
- Prat met ons **in jouw taal:** 0800 60 160 (FREE OF CHARGE)
- Download deze brief **in jouw taal:** scan the QR-code

Met vriendelijke groete,

Dr. Patrick Martens
Directeur Centrum voor Kankeropsporing (CvKO)

BEVOLKINGS ONDERZOEK.BE | VZW CENTRUM VOOR KANKEROPSPORING | ADMINISTRATIEVE ZETEL RUDDERSHOVE 4, 8000 BRUGGE | WWW.BEVOLKINGSONDERZOEK.BE INFO@BEVOLKINGSONDERZOEK.BE | CvKO CENTRUM VOOR KANKEROPSPORING

Het CvKO kreeg de toestemming van de Privacycommissie om uw gegevens op te vragen bij de desbetreffende instanties en te gebruiken in het kader van dit bevolkingsonderzoek. Deze Commissie waakt er over dat elk gebruik ervan verloopt met respect voor de Privacywetgeving.

Picture S4 – Interim draft of a reminder letter for the breast cancer screening program, tailored to the needs of underserved women living in Flanders (English translation, 2023)

BEVOLKINGS
ONDERZOEK
BORSTKANKER

Vlaanderen
is zorg

Your personal code:

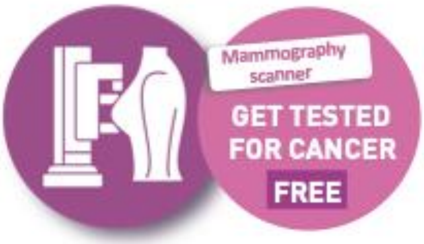

To

Dear

We want to test you for breast cancer free of charge. The mammography machine helps see breast cancer early, before you feel anything in your breast. This is important for your health.

Many women in Flanders, **more than 200,000 each year,** have been tested.
Do you want to be tested too? **GO TO THE APPOINTMENT!**

Go to this appointment:

[00/00/0000, at 00:00]

Mammography unit: [MU NAME]

[MU STREET NUMBER P.O. BOX, POSTCODE TOWN]

Bring these things:

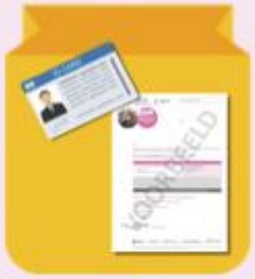

- your ID card
- this letter

THE TEST IS FREE OF CHARGE

Do you have questions about how the scanner works? Or do you want to make a new appointment?

- Read the brochure in the envelope
- Discuss this with your GP
- E-mail info@bevolkingsonderzoek.be

- Talk with us **in your language:** 0800 60 160 (FREE OF CHARGE)

- Download this letter **in your language:** scan the QR-code

Kind regards,

Dr Patrick Martens
Director, Centre for Cancer Detection (CvKO)

BEVOLKINGS ONDERZOEK.BE
VZW CENTRUM VOOR KANKEROPSPORING
ADMINISTRATIEVE ZETEL RIJDERSHOVE 4, 8000 BRUGGE
WWW.BEVOLKINGSONDERZOEK.BE
INFO@BEVOLKINGSONDERZOEK.BE
CvKO CENTRUM VOOR KANKEROPSPORING

Het CvKO kreeg de toestemming van de Privacycommissie om uw gegevens op te vragen bij de desbetreffende instanties en te gebruiken in het kader van dit bevolkingsonderzoek. Deze Commissie waakt er over dat elk gebruik ervan verloopt met respect voor de Privacywetgeving.

Picture S5 – Final reminder letter for the breast cancer screening program, tailored to the needs of underserved women living in Flanders (Dutch version, 2023)

LAAT JE CONTROLEREN OP BORSTKANKER GRATIS

DEZE BRIEF IS BELANGRIJK!

Je persoonlijke code:

Aan

Spreek je geen Nederlands? Download deze brief in een andere taal.
Deutsch, English, Español, Français, Italiano, Русский,
Română, Shqip, Türkçe, فارسی , العَرَبِيَّة , 中文.

SCAN DE QR-CODE

Beste

Je kunt je gratis laten controleren op borstkanker. Dit is **belangrijk** voor je gezondheid.
Wil jij ook gecontroleerd worden? Kom naar jouw afspraak.

Jouw afspraak	Wat breng je mee?
Datum en uur:<br><br>Adres:	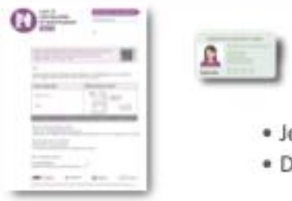<br>• Je ID-kaart<br>• Deze brief

De controle is GRATIS vanaf 50 jaar als je bij een Belgische mutualiteit bent

Wil je een nieuwe afspraak maken?
- Mail naar: info@bevolkingsonderzoek.be
- Bel gratis naar: 0800 60 160 (maandag-donderdag 8-12u en 13-17u, vrijdag 8-12u en 13-16u)

Als je al een nieuwe afspraak hebt gemaakt, moet je met deze brief geen rekening houden.

Heb je vragen over de controle?
- Bespreek dit met je huisdokter
- Surf naar: www.borstkanker.bevolkingsonderzoek.be

Met vriendelijke groeten,

Dr. Patrick Martens
Directeur Centrum voor Kankeropsporing

BEVOLKINGS ONDERZOEK BORSTKANKER · Vlaanderen · BEVOLKINGS ONDERZOEK.BE · CvKO CENTRUM VOOR KANKEROPSPORING

Het CvKO kreeg de toestemming van de Privacycommissie om uw gegevens op te vragen bij de desbetreffende instanties en te gebruiken in het kader van dit bevolkingsonderzoek. Deze Commissie waakt er over dat elk gebruik ervan verloopt met respect voor de Privacywetgeving. VZW Centrum voor Kankeropsporing. Administratieve Zetel Ruddershove 4, 8000 Brugge.

Picture S6 – Final reminder letter for the breast cancer screening program, tailored to the needs of underserved women living in Flanders (English translation, 2023)

GET CHECKED FOR BREAST CANCER

FREE OF CHARGE

THIS LETTER IS IMPORTANT!

Your personal code:

To

Don't speak Dutch? Download this letter in another language.

Deutsch, English, Español, Française, Italiano, Русский, Română, Shqipja, Türkçe, فارسی , العَرَبِيَّة , 中文.

SCAN THE QR CODE

Dear

You can get checked for breast cancer free of charge. This is **important** for your health. Would you like to be checked? Come to your appointment.

Your appointment	What do you bring?
Date and time:<br><br>Address:	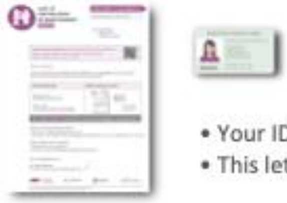<br>• Your ID card<br>• This letter

The check-up is FREE from the age of 50 if you are a member of a Belgian health insurance company

Would you like to make a new appointment?

- E-mail : info@bevolkingsonderzoek.be
- Call toll-free: 0800 60 160 (Monday-Thursday 8-12am and 1-5pm, Friday 8-12am and 1-4pm)

Do you have questions about the check-up?

- Discuss them with your family doctor.
- Visit: www.borstkanker.bevolkingsonderzoek.be

Kind regards,

Dr. Patrick Martens
Director, Center for Cancer Screening

BEVOLKINGS ONDERZOEK BORSTKANKER — Vlaanderen is zorg — BEVOLKINGS ONDERZOEK.BE — CvKO CENTRUM VOOR KANKEROPSPORING ANTWERPEN BRUGGE BRUSSEL GENT LEUVEN

Het CvKO kreeg de toestemming van de Privacycommissie om uw gegevens op te vragen bij de desbetreffende instanties en te gebruiken in het kader van dit bevolkingsonderzoek. Deze Commissie waakt er over dat elk gebruik ervan verloopt met respect voor de Privacywetgeving. VZW Centrum voor Kankeropsporing. Administratieve Zetel Ruddershove 4, 8000 Brugge.
